# Management of heterotopic pregnancy: clinical analysis of sixty-five cases from a single institution

**DOI:** 10.3389/fmed.2023.1166446

**Published:** 2023-05-10

**Authors:** Feng Ge, Wei Ding, Kun Zhao, Pengpeng Qu

**Affiliations:** ^1^Clinical School of Obstetrics and Gynecology Center, Tianjin Medical University, Tianjin, China; ^2^Department of Gynecological Oncology, Tianjin Central Hospital of Gynecology and Obstetrics, Tianjin, China

**Keywords:** heterotopic pregnancy, treatment, pregnancy outcome, laparoscopy, assisted reproductive technology

## Abstract

**Objective:**

This retrospective study aims to analyze the influence of different treatment modalities on viable intrauterine pregnancy and to summarize the clinical features of heterotopic pregnancy (HP) patients.

**Material and methods:**

All patients diagnosed with HP between January 2012 and December 2022 in Tianjin Central Obstetrics and Gynecology Hospital were reviewed retrospectively.

**Results:**

This study diagnosed 65 patients using transvaginal ultrasound (TVS), including two cases of natural pregnancy, seven cases of ovulation induction pregnancy, and 56 cases after *in vitro* fertilization and embryo transfer (IVF-ET). The gestational age was 50.2 ± 13.0 days at the time of diagnosis. The most frequent manifestations were abdominal pain (61.5%) and vaginal bleeding (55.4%), while 11 patients (16.9%) had no symptoms before the diagnosis. The primary treatment was expectant and surgical management, including laparotomy and laparoscopic surgery. In the expectant management group, four patients were transferred to surgery due to rupture of ectopic pregnancy or ectopic pregnancy mass gradually enlarged. In the surgical management group, 53 patients underwent laparoscopic surgery, and six underwent laparotomy. The laparoscopic group's mean operation time was 51.3 ± 14.2 min (range: 15–140 min), and the median intraoperative blood loss was 20 mL (range 5–200 mL). In contrast, the laparotomy group's mean operation time was 80.0 ± 25.3 min (range 50–120 min), and the median intraoperative blood loss was 22.5 mL (range 20–50 mL). Four patients had postoperative abortions. Sixty-one newborns had no birth abnormalities, and no developmental malformations were discovered after a median follow-up of 32 months.

**Conclusion:**

Expectant treatment has a high failure rate in HP, and laparoscopic surgery is a safe and effective treatment for removing ectopic pregnancy without increasing the risk of abortion or newborn birth defects.

## Introduction

Heterotopic pregnancy (HP) is a pathological pregnancy in which intrauterine and ectopic pregnancy coexist (1). Most reported ectopic gestational sacs of HP are located in the fallopian tube. Few are in the corner, with rare reports of cesarean section scar sites, ovary, abdominal cavity, and cervix. HP incidence is one in 30,000 normal gestations (2). The incidence arises from 0.09 to 1% with assisted reproductive technology (ART) application, which may be related to the history of fallopian tube disease (3). Additionally, pelvic surgery or inflammation may be a risk factor for HP. The HP treatment typically includes expectant therapy, drug therapy, surgery, and interventional therapy (4). However, intrauterine fetuses are extremely valuable for most patients. The primary goal of HP treatment is to ensure the safety of pregnant women while minimizing intrauterine pregnancy damage. Therefore, early diagnosis and appropriate treatment can reduce complications, such as fallopian tube rupture, shock, and blood transfusion.

This retrospective study aims to analyze the influence of different treatment modalities on viable intrauterine pregnancy and to summarize our department's experiences in HP management.

## Materials and methods

Sixty-five patients were diagnosed as HP using TVS or surgical pathology in Tianjin Central Obstetrics and Gynecology Hospital between January 2012 and December 2022. Ultrasonic diagnostic criteria for HP: a visible intrauterine pregnancy sac (i) an inhomogeneous adjunct mass or a mass adjacent to the ovary but relatively separable from the ovary; (ii) an ectopic pregnancy sac regarded as a hyperechoic ring; (iii) Yolk sac and/or embryo (with or without fetal heart) in extrauterine gestation sac. Exclusion criteria for this study involve patients diagnosed with intrauterine pregnancy who underwent complete curettage of the uterine cavity and removal of ectopic pregnancy lesions before surgery; histopathological examination of suspicious pregnancy tissue revealed the absence of villi tissue. Two patients lost follow-up of pregnancy outcomes. All medical records and sonographic images were collected and reviewed to exclude misdiagnosis. All clinical data used for research purposes were approved in writing by the patients and approved by the Ethics Committee of Tianjin Central Obstetrics and Gynecology Hospital.

All patients received expectant treatment and surgery. The choice depends on the patient's clinical symptoms, hemodynamic parameters, repeat TVS results, and patient selection. Surgical treatment is recommended in the following cases: (i) Yolk sac echo or primitive cardiac duct pulsation observed in abnormal echo area; (ii) Abnormal echo area diameter > 3 cm; (iii) Abnormal echo area gradually increased, and the diameter was > 3 cm; (iv) Presenting internal bleeding or progressive decreased of hemoglobin. Combined thoracolepidural anesthesia was used for exploratory laparotomy, while general intravenous anesthesia was used for laparoscopic surgery, maintaining CO_2_ pneumoperitoneum pressure below 12 mmHg. Initially, the hematocele was removed from the pelvic cavity, followed by the localization of the ectopic pregnancy mass for surgical procedures. If the pregnancy was tubal, the affected side of the fallopian tube was resected; if the pregnancy was pelvic, the pregnancy material was removed. The uterine wound was sutured with 2-0 absorbable thread and washed with 0.9% sodium chloride solution during the operation. Oral dydrogesterone was administered after surgery, and antibiotics were not routinely administered to prevent infection. If the patient recovered, she could be discharged 3 days after surgery with no abnormalities in serum β-human chorionic gonadotropin (β-hCG), progesterone, and ultrasonography. Each patient was contacted by phone to inquire about the progress of their pregnancy. The follow-up endpoint was December 2022.

SPSS 20.0 software was used for statistical analysis. The mean ± standard deviation was used to describe quantitative data with a normal distribution, while the *T*-test or rank-sum test was employed to compare groups. The quantitative data with non-normal distribution were described using median and quartile spacing, and inter-group comparisons were performed using the rank-sum test. Groups were compared using frequency and composition of qualitative data, the chi-square test, or Fisher's exact probability method.

## Results

### Clinical characteristics of HP

Table 1 presents the clinical characteristics of 65 HP patients. The HP patient included two cases of natural pregnancy, seven cases of ovulation induction pregnancy, and 56 cases of pregnancy after IVF-ET. The age ranged from 20 to 41 years, with an average of 30.5 ± 4.4 years. Eleven cases had a history of ectopic pregnancy, including seven cases with a history of ectopic pregnancy surgery. Fifty-four patients (83.1%) were diagnosed with HP based on clinical symptoms, including severe abdominal pain and/or vaginal bleeding. Twenty-eight patients (43.1%) had hemoperitoneum, and 10 (15.4%) developed hypovolemic shock, requiring a blood transfusion. Eleven patients (16.9%) were asymptomatic and diagnosed with HP during a routine ultrasound examination.

**Table 1:** General characteristics and clinical outcomes of HP patients.

**Variables**	**No. (%) of patients**
Age, mean ± SD (range), yr	30.5 ± 4.4
History of abortion, *n* (%)	
0	39 (60.0)
1–2	17 (26.2)
≥3	9 (13.8)
History of pelvic surgery, *n* (%)	
Tubal surgery	11 (16.9)
Non-tubal surgery	7 (10.8)
No surgery	47 (72.3)
Type of infertility, *n* (%)	63
Primary	39 (61.9)
Secondary	24 (38.1)
Method of IVF-ET, *n* (%)	57
Fresh non-donor embryo	22 (38.6)
Frozen-thawed embryo	35 (61.4)
Number of transferred embryos, *n* (%)	63
1	1 (1.6)
2	44 (69.8)
3	18 (28.6)
Clinical manifestations, *n* (%)	
Asymptomatic	11 (16.9)
Abdominal pain or/and vaginal bleeding	54 (83.1)
Hypovolemic shock	10 (15.4)
Gestational age at diagnosis, mean ± SD (range), d	50.2 ± 13.0
Site of ectopic pregnancy, *n* (%)	59
Tubal	51 (86.4)
Interstitial	4 (6.8)
Corner	4 (6.8)
Management of HP, *n* (%)	
Expectant management	10 (15.4)
Surgical management	55 (84.6)
Blood transfusion, *n* (%)	
Yes	10 (15.4)
No	55 (84.6)
Clinical outcomes, *n* (%)	
Term delivery	58 (89.2)
Preterm delivery	3 (4.6)
Abortion	4 (6.2)
Mode of delivery, *n* (%)	
Vaginal delivery	24 (39.3)
Cesarean section	37 (60
Apgar's score	
<10	4 (6.6)
10	57 (93.4)

The serum β-hCG ranged from 1,066 to 124,736 mIU/mL (median: 42,865.5 mIU/mL). Fifty-five HP patients received immediate surgical treatment, while 10 insisted on the expectant treatment. Two patients with expectant treatment ruptured ectopic pregnancy, with 200 mL and 2,800 mL pelvic hematocele during the operation. Two patients undergoing expectant treatment experienced ectopic pregnancy rupture, with pelvic hematocele volumes of 200 and 2,800 mL, respectively, during the operation. Therefore, 59 patients received surgical treatment, with 53 undergoing laparoscopic surgery and six undergoing laparotomy. The laparoscopic group's mean operation time was 51.3 ± 14.2 min (range 15–140 min), and the median intraoperative blood loss was 20 mL (range 5–200 mL), while the laparotomy group's mean operation time was 80.0 ± 25.3 min (range 50–120 min), and the median intraoperative blood loss was 22.5 mL (range 20–50 mL). Salpingectomy was performed in 56 cases, including 46 (82.1%) ectopic pregnancies located in the ampulla, five (8.9%) in the isthmus, four (7.1%) in the interstitium, and four (7.1%) in the corner, whereas partial oophorectomy was performed in one case of ovarian pregnancy. Resection of ectopic pregnancy was completed in one case of pelvic pregnancy. One patient had previously undergone salpingectomy for right fallopian tube pregnancy. After embryo transfer, ultrasound suggested a right fallopian tube stump pregnancy, and laparoscopy revealed the stump pregnancy. After the operation, no antibiotics were administered, and all patients recovered.

### Pregnancy outcomes

Four of the 65 patients (6.15%) experienced an abortion, including two patients before 12 weeks of pregnancy, one at 15 weeks, and one with a fetal arrest at 23 weeks.

There were 58 (95.08%) full-term deliveries and three (4.92%) premature deliveries, including one cesarean section due to pregnancy-induced hypertension at 31 weeks of gestation and two cases of premature rupture of membranes at 36 weeks of gestation. There were 37 (60.66%) cesarean-section pregnancies and 24 (39.34%) vaginal deliveries. Apgar scores ranged from 7 to 10, with 10 being the average. Sixty-one newborns had no birth abnormalities, and no developmental malformations were discovered after a median follow-up of 32 months.

## Discussion

The ectopic gestational sac of HP can be located most frequently in the fallopian tube, uterine corner, ovary, cervix, abdominal cavity, or uterus scar incision. A history of ectopic pregnancy or pelvic inflammatory disease is considered a high-risk factor for HP (2, 3). In our study, 94.7% of ectopic gestational HP located in the fallopian tube, and 11 patients had a previous ectopic pregnancy history, similar to literature reports. Additionally, we discovered that tubal stump pregnancy could occur after tubal resection with IVF-ET. Therefore, HP cannot be avoided entirely, even if the fallopian tube is removed or ligated. In our study, 55 patients underwent hysteroscopy. Iatrogenic uterine injury caused by hysteroscopy has been previously reported as a risk factor for uterine rupture, but whether it leads to HP remains uncertain (5). These patients should receive increased attention for ectopic pregnancies at unusual sites.

Clinical manifestations of HP are untypical; common presentations include vaginal bleeding and acute abdominal pain, which were similar to ectopic pregnancy. However, these symptoms can also be observed in intrauterine pregnancies, and easy to leading missed diagnosis. HP is frequently misdiagnosed as threatened abortion due to the simultaneous existence of intrauterine pregnancy. Unless there is acute abdominal pain or shock associated with a ruptured ectopic pregnancy, one report points out that 27.1~50% of patients have no clinical symptoms, increasing the difficulty of diagnosis (6). Our result revealed that the most common symptoms were abdominal pain and vaginal bleeding, but 16.9% (11/65) of patients had no symptoms, similar to the results of previous studies. Therefore, patients with vaginal bleeding or abdominal pain should be alert to HP occurrence for intrauterine pregnancy after ART. The frequency of ultrasound examinations should be increased to help with early HP diagnosis.

Continuous determination of serum β-hCG is essential for diagnosing ectopic pregnancy, but it is meaningless for diagnosing HP due to intrauterine pregnancy (7). Ultrasound examination is necessary for the safety of intrauterine pregnancy and the accuracy of ectopic pregnancy diagnosis in HP patients. The accuracy rate of preoperative ultrasound diagnosis was 95.06% (8). In our study, only 56.9% of patients were suspected of suffering from HP during the initial ultrasound examination, with the remaining patients presenting with complaint symptoms. Ultrasound doctors previously only paid attention to the scan of intrauterine pregnancy due to the low incidence of HP, resulting in many missed diagnoses. However, as the number of ART patients increased, even if the ultrasound doctors discovered an intrauterine pregnancy, it was still necessary to carefully scan the bilateral adnexal area.

Pelvic bleeding caused by ectopic pregnancy rupture is the primary cause of death in pregnant women. It is also important to consider patient condition monitoring and decision operation. The time of menopause, serum β-hCG value, size of the external uterine mass, and history of ectopic pregnancy may be associated with tubal pregnancy rupture. The interstitial/corner musculature of the fallopian tube is thicker and can support embryo growth for longer than the non-interstitial part. Rupture typically occurs between 12 and 16 weeks of gestation (9). In our study, the earliest rupture occurred on the 37th day after IVF-ET, and the latest rupture occurred on the 80th day, which was similar to the pattern of interstitial/corner pregnancy rupture caused by natural conception, suggesting that we should treat ectopic pregnancy at the special site after ART carefully to avoid serious complications.

Assisted reproductive experts recommend that the treatment principle for HP is to remove the ectopic pregnancy while minimizing the impact on the intrauterine pregnancy, but there is still no consensus. The primary therapies include expectant, drug, interventional, and surgical treatments. The premise of expectant and drug treatment is that the patient's vital signs are stable, the ectopic pregnancy has not ruptured, and current drug use can have adverse effects on intrauterine embryos.

A study discovered that an ultrasound-guided reduction is preferable to surgery for HP patients to preserve the intrauterine pregnancy. Aspiration or local injection of methotrexate, potassium chloride, and hypertonic sugar solution facilitated the ultrasound-guided reduction (10). It has the advantages of less trauma and anesthetic drug influence on fetuses. After ultrasound-guided reduction, successful cesarean-section deliveries have also been reported among HP patients (11, 12). However, some doctors are concerned that methotrexate may increase the risk of fetal abnormalities. In Liu's report, five cases of ultrasound-guided local injection of anhydrous ethanol to treat HP, with the injection dose of 1.0–2.5 mL. Among the patients, four pregnancies were delivered successfully, and one resulted in a miscarriage due to an ectopic pregnancy sac rupture (13). However, a study revealed that the probability of intraperitoneal bleeding after ultrasound-guided reduction is higher than during laparoscopic surgery (14). Therefore, clinicians should inform patients of the risks of ultrasound-guided reduction to avoid threats to maternal and intrauterine fetal health. Interventional therapy requires highly selective specific vascular embolization. However, vascular embolization alone is ineffective due to the presence of vascular communication branches and frequently requires combination with drug therapy, limiting its clinical application (15).

Surgery is still the primary treatment for HP, and no adverse effects on the fetus have been reported. According to studies, there is no statistically significant difference between the impact of laparotomy and laparoscopic surgery on the abortion rate of intrauterine pregnancy (16). Current reports have proved that laparoscopic surgery does not increase the risk of maternal and infant adverse effects during pregnancy, and it has the advantages of quicker postoperative recovery, enabling faster postoperative deambulation and return to regular activity compared to laparotomy (17). Our study revealed that the postoperative abortion rate of patients undergoing laparotomy and laparoscopic surgery was 5.9 and 16.7%, respectively, with no statistically significant difference between the two groups. However, some articles pointed out that emergency surgical intervention for ovarian torsion or spontaneous rupture with hematoperitoneum increases the risk of abortion and preterm delivery compared with patients undergoing elective surgery (17). According to the guidelines of British Society of Gynecological Endoscopy and the Royal College of Obstetricians and Gynecologists (RCOG) published in 2019, it is recommended to use 20–25 mmHg pressure when establishing pneumoperitoneum, and it is safe for the fetus to maintain the pressure at 12 mmHg during the operation. Hasson's puncture can determine the location of the first puncture hole according to the fundus uteri height, lowering the risk of uterine injury (18). The use of anesthetic drugs during surgery is another area where patients and doctors are concerned about possible adverse effects on the fetus *in utero*. In our study, all patients were under general anesthesia, and none of the newborns occurred birth defects. Recently, according to general surgeons' experience, the combination of minimally invasive surgery and regional anesthesia appeared to increase laparoscopic procedure advantages and emergency abdominal surgery under regional anesthesia during pregnancy and successful continuation of pregnancy have been reported in the literature (19, 20). However, it is still controversial whether anesthetic drugs have an effect on the long-term psychological and physiological development of the fetus *in utero*.

There is still no consensus on the standard of expectant treatment for HP patients. A comprehensive evaluation should be carried out based on the patient's willingness and compliance. There are no large-scale reports of expected HP treatment. Li et al. reported 50 cases of HP and discovered that among the 20 patients who received expectant treatment, four cases of ectopic pregnancy ruptured, six cases needed surgical treatment, and one case miscarried due to fever during expectant treatment. They concluded that the outcome of intrauterine pregnancy adopting expectant treatment was the worst (4). In our study, two patients out of 10 who chose expectant treatment had a rupture of ectopic pregnancy. Two patients were transferred to surgical management, suggesting that expectant treatment should be carefully selected for HP patients.

There have been no large-scale studies on whether the newborn of HP patients has defects. Studies have demonstrated that surgical treatment does not increase the risk of birth defects at all stages of pregnancy (21). Our study discovered no neonatal defects. According to studies, the rupture of an ectopic pregnancy causing intraperitoneal massive bleeding results in hypovolemic shock in the mother and severe ischemic brain injury in the intrauterine fetus, leading to the development of fetal cerebral palsy (22, 23). During the follow-up period of our study, no children developed dysplasia. That the incidence of fetal ischemic brain injury caused by maternal hypotension must be minimized during treatment.

## Conclusion

HP incidence also increases annually with ART application. Combining clinical manifestations and ultrasonography is the primary method for early HP diagnosis. Clinicians should select the expected treatment with care due to the high risk of rupture or surgical transfer. Expectant treatment has a high failure rate in HP, and laparoscopic surgery is a safe and effective treatment for removing ectopic pregnancy without increasing the risk of abortion or newborn birth defects.

## Data availability statement

The datasets presented in this study can be found in online repositories. The names of the repository/repositories and accession number(s) can be found in the article/supplementary material.

## Ethics statement

The study was conducted in accordance with the principles of the Declaration of Helsinki, and the study protocol was approved by the Ethics Committee of Tianjin Central Hospital of Gynecology Obstetrics. We obtained patient consent before the study. Written informed consent was obtained from the participant for the publication.

## Author contributions

PQ and FG designed and organized the study. WD collected and prepared the data of patients. FG and WD wrote the first article draft. KZ supervised the study. All authors contributed to the article and approved the submitted version.
